# Benefits and Challenges of Rare Genetic Variation in Alzheimer’s Disease

**DOI:** 10.1007/s40142-019-0161-5

**Published:** 2019-02-01

**Authors:** Detelina Grozeva, Salha Saad, Georgina E. Menzies, Rebecca Sims

**Affiliations:** 1 Division of Psychological Medicine and Clinical Neuroscience, MRC Centre for Neuropsychiatric Genetics and Genomics, School of Medicine, Cardiff University, Cardiff, UK; 2 UK Dementia Research Institute at Cardiff, School of Medicine, Cardiff University, Cardiff, UK

**Keywords:** Alzheimer’s disease, Genetics, Susceptibility, Rare variants

## Abstract

**Purpose of Review:**

It is well established that sporadic Alzheimer’s disease (AD) is polygenic with common and rare genetic variation alongside environmental factors contributing to disease. Here, we review our current understanding of the genetic architecture of disease, paying specific attention to rare susceptibility variants, and explore some of the limitations in rare variant detection and analysis.

**Recent Findings:**

Rare variation has been shown to robustly associate with disease. These include potentially damaging and loss of function mutations that are easily modelled in silico, in vitro and in vivo, and represent potentially druggable targets. A number of risk genes, including *TREM2*, *SORL1* and *ABCA7* show multiple independent associations suggesting that they may influence disease via multiple mechanisms. With transcriptional regulation, inflammatory response and modification of protein production suggested to be of primary importance.

**Summary:**

We are at the beginning of our journey of rare variant detection in AD. Whole exome sequencing has been the predominant technology of choice. While fruitful, this has introduced a number of challenges with regard to data integration. Ultimately the future of disease-associated rare variant identification lies in whole genome sequencing projects that will allow the testing of the full range of genomic variation.

## Introduction

Alzheimer’s disease (AD) is a devastating and progressive neurodegenerative disease that is estimated to account for up to 80% of all dementia cases, meaning there are over 37 million AD sufferers world-wide. With an ageing population, the incidence of AD is expected to rise exponentially, and with no effective prevention or treatment, dementia is now one of the world’s greatest public health issues.

It is well established that while AD has some symptoms common to all sufferers, aetiologically, there are at least two different forms of disease. A small number of individuals (~1%) carry a disease-causing mutation within the *APP*, *PSEN1* or *PSEN2* genes. These Mendelian forms of disease underpin the amyloid cascade hypothesis of AD. The hypothesis claims the misprocessing of β-amyloid (Aβ) and its deposition as the primary causal event in AD pathogenesis [1]. However, the failures of clinical trials focussed on Aβ pathology suggest that this hypothesis may only relate to Mendelian forms of disease. Conversely, these tested therapeutics may only be effective in the prodromal stages of AD, rather than the symptomatic phase when participants for clinical trials are recruited [2, 3]. Undoubtedly, the amyloid cascade is part of a complex interplay of influences on disease development that includes tau and immunity/inflammation.

Other forms of disease are seen to segregate, although not completely, in families where there are no fully penetrant causative mutations (heritability estimates of over 90%) [4]. Non-familial, sporadic AD is a highly heritable (estimates of 58–79%) [5] and polygenic disorder [6], with over 40 risk loci reliably established [3]. The majority of the identified loci are common (minor allele frequency > 1%) with small effect sizes and reside in non-coding parts of the genome. The identification of these common loci has greatly improved our understanding of the underlying biology of disease, highlighting immunity, endocytosis, cholesterol metabolism, protein ubiquitination and more recently Aβ processing [7]. However, the identified common variants do not pinpoint protein coding changes for direct modelling, nor do they explain the estimated disease heritability. In fact, the common variants known to associate with AD are thought to account for less than half of the genetic liability for disease [8]. In recent years, and as genotyping and sequencing technologies have advanced, the field has focused on the identification of rare variation for disease. Here, we will focus on these discoveries, the technologies that enabled their discovery and the challenges of working with big data for rare variant detection.

## Identified Common Risk Loci

Risk for non-familial forms of AD is inferred by both environmental and genetic factors, with common and rare variation involved in non-Mendelian disease aetiology. *Apolipoprotein E* (*APOE*) on chromosome 19 was the first and remains the strongest genetic risk factor for AD [9]. *APOE* encodes three isoforms of the protein, ε2, ε3 and ε4. Disease risk is increased in carriers of the ε4 allele, in a dose-dependent manner, with a threefold increase in ε4 heterozyogotes (ApoE ε3/ε4), and a 15-fold increase in ε4 homozygotes (ApoE ε4/ε4). The ε2 allele is thought to confer a small protective effect [10, 11].

Over the past 9 years, genome-wide association studies (GWAS) in case-control cohorts of tens of thousands of individuals have identified nearly 40 common genome-wide significant risk loci. The identified susceptibility variants generally have small effect sizes (odds ratios ~ 1.2) and are often found in intergenic or intronic regions (therefore termed locus rather than gene) making it difficult to pinpoint which gene has a functional effect. The *CLU*, *PICALM* and *CR1* loci [12, 13] were identified in 2009 in two back-to-back publications. Subsequent publications have identified *BIN1*, *EPHA1, MS4A, CD2AP*, *ABCA7*, *HLA-DRB5/HLA-DRB1*, *PTK2B*, *SORL1*, *SLC24A4*-*RIN3, INPP5D, MEF2C, NME8,* *ZCWPW1, CELF1, FERMT2* and *CASS4* as risk loci for sporadic AD [14–16]. This success can be largely attributed to the extensive collaboration across four genetic consortia; Genetic and Environmental Risk in AD (GERAD), European AD Initiative (EADI), Cohorts for Heart and Aging Research in Genomic Epidemiology (CHARGE) and AD Genetics Consortium (ADGC), badged together as the International Genomics of Alzheimer’s Project (IGAP). In addition, genome-wide gene-wide analyses identified five novel genome-wide significant loci, *TP53INP1*, *IGHV1*–*67,* [17] *PPARGC1A*, *RORA* and *ZNF423* [18] using the IGAP dataset. Building upon the initial IGAP dataset, *TRIP4* [19], *ECHDC3* [7, 20], *IQCK*, *ACE*, *ADAM10* and *ADAMTS1* [7] have more recently been reported as genome-wide susceptibility loci.

A novel approach to AD risk variant detection has been to infer AD diagnosis based on reports of parental history of dementia in the UK Biobank dataset [21•, 22]. Although this approach lacks power and introduces diagnostic noise, it proved informative when combined with clinically defined AD cohorts. Combination of UK Biobank data with additional datasets identified association for AD risk with variants in *KAT8* [21•], *HESX1*, *CLNK/HS3ST1*, *CNTNAP2*, *APH1B*, *ALPK2* and *LOC388553* loci [22•].

It is estimated that a substantial proportion (approximately 60%) [23, 24] of the genetic variance of sporadic AD is not accounted for by *APOE* or the common genome-wide associated loci. GWAS in other complex traits suggest that more powerful GWAS will identify further additional associations [25]. However, a substantial proportion of the ‘missing heritability’ of AD is likely to be accounted for by rare and low frequency variation of small to moderate effect.

## Identified Rare Risk Loci

A flurry of recent reports has confirmed that a proportion of AD is explained by rare variation with larger effect sizes than normally seen with common variation. Such variants point to the involvement of novel genes in the pathophysiology of disease and importantly highlight protein-coding changes that are novel therapeutic targets.

To date, four genes have been identified showing replicable genome-wide significant association with disease. In 2013, two groups identified p.Arg47His (R47H) within the triggering receptor expressed on myeloid cells 2 (*TREM2*) gene as a lateonset AD risk variant; the independent studies were published in back-to-back publications in the New England Journal of Medicine [26, 27]. Both studies utilised a multi-stage study design. Guerreiro and colleagues [27] undertook whole exome sequencing (WES) and Sanger sequencing in a discovery cohort of 1092 AD cases and 1107 controls, before confirming their identified association through additional independent genotyping and meta-analysis of imputed GWAS data. Simultaneously, Jonsson and colleagues [26] identified the same *TREM2* variant through whole genome sequencing (WGS) of 2261 Icelandic participants and reported that the R47H variant significantly associated with risk of AD in a largely Scandinavian population. Replication of their findings was achieved through additional genotyping of independent cohorts and imputed datasets from Europe and the USA, with meta-analysis of the datasets showing association at the genome-wide level (odds ratio, 2.90; 95% CI, 2.16 to 3.91; *P* = 2.1 × 10 ^−12^) [26]. The association at *TREM2* p.(Arg47His) and AD has been replicated in multiple populations of European descent [28–35, 36•, 37, 38], and although the risk-effect sizes vary by cohort, cumulatively, the results suggest that *TREM2* R47H is the largest genetic effector of sporadic AD after *APOE* ε4.

A second *TREM2* variant that had previously shown suggestive evidence for association with disease [39, 40•, 41] was shown to increase risk for sporadic AD at the genome-wide significance level via an exome-chip microarray study [36•]. The Illumina exome-chip was designed as an intermediate experiment between current genotyping arrays, which focus on relatively common variants, and exome sequencing of very large numbers of samples. The array contains over a quarter of a million variants identified from WGS and WES data of over 12,000 individuals. We and others showed a rare coding mutation at *TREM2* p.Arg62His (R62H) increased risk for disease independently of the R47H *TREM2* mutation with an odds ratio of 1.67 [36•]. In the same publication, we identified novel association within two additional genes, *phospholipase C gamma 2* (*PLCG2*) and *ABI Family Member 3* (*ABI3*). The *PLCG2* variant p.Pro522Arg (P522R) shows a protective effect against disease, while the *ABI3* variant p.Ser209Phe (S209F) increased disease risk [36•]. AD risk inferred by *TREM2* does not appear to be population-specific with association reported in European [26, 27, 36•], African American [42] and Asian [43] populations. However, a number of studies show conflicting data with the *TREM2* R47H variant not significantly associated with AD risk in an African-American cohort [38], and four studies failing to detect the R47H variant in Chinese subjects [44–47]. In one study of Japanese subjects, the R47H variant was extremely rare (minor allele frequency < 0.006) and no association was found with AD.

In 2012, Jonsson and colleagues [39] showed, for the first time in sporadic AD, an association with the *amyloid precursor protein* (*APP*) gene that causes familial forms of AD. The identified protein-coding change p.Ala673Thr (A673T) was identified in WGS data from 1795 Icelanders and was shown to protect against disease, and cognitive decline in elderly non-diseased participants [39]. The protein change is thought to reduce β-cleavage of APP with approximately 40% reduction in the formation of amyloidogenic peptides in vitro.

Exome-wide significant association with sporadic disease was recently identified in the AD Sequencing Project (ADSP). Novel single nucleotide variant (SNV) association with disease was identified at the *IGHG3* (an immunoglobulin gene whose antibodies interact with β-amyloid) and *AC099552.4* (a long non-coding RNA) genes, while exome-wide gene-wide association was identified at the *ZNF655* (zinc-finger protein) gene [40•]. These newly discovered genes point to the important role of transcriptional regulation in AD pathogenesis and add further support to the role of inflammatory response and modification of protein production in disease biology [40•]. Analysis of the ADSP data to examine the contribution to disease across dementia genes and clinically diagnosed AD identified rare pathogenic variants within *ARSA*, *CSF1R* and *GRN*, along with candidate variants in *GRN* and *CHMP2B*. A further independent casecontrol study provided evidence of association between variants in *TREM2*, *APOE*, *ARSA*, *CSF1R*, *PSEN1* and *MAPT* and risk of AD [41]. Interestingly, the ADSP also identified a number of rare disease-associated variants within loci known to harbour common variants associated with sporadic AD, including *ABCA7* and *SORL1*. [48–51]. Bellenguez and colleagues [50] also observed that variants in *SORL1*, *ABCA7*, *TREM2* associated with AD. More specifically, the authors identified an exome-wide significant association between early onset AD risk and rare variants in all three genes. The authors estimated that the associated variants contributed equally to the heritability of early onset AD, and each explains between 1.1 and 1.5% of sporadic early onset AD heritability [50]. Further evidence for the role of *SORL1* in AD aetiology was provided by Holstege and colleagues [52], who observed that unique protein-truncating variants in *SORL1* occurred exclusively in a substantial proportion of AD cases. Variants in *ABCA7* have been identified to influence disease risk both across ethnic populations [15], and in an ethnic specific manner, with a frameshift deletion identified in African American and Caribbean Hispanic populations, but not a non-Hispanic White population [53].

Another novel gene with rare coding variants observed to segregate in an autosomal-dominant way with AD is *UNC5C* [54]. Wetzel-Smith et al. proposed that the variants in the gene could contribute to developing the disease by increasing susceptibility to neuronal cell death in vulnerable regions of the brain in patients with AD. Based on a family-based study and further replication, Cruchaga et al. observed that low frequency variants in *PLD3* were enriched in individuals with AD compared to healthy controls. Furthermore, the authors also showed that *PLD3* was involved in amyloid-β precursor protein processing and was overexpressed in brain tissue from patients with AD [55]. However, at present, the statistical evidence for association with sporadic disease at these gene is not robust, with *PLD3* seeming to have a greater influence on familial forms of disease [32, 56–58].

Other notable studies identifying rare variant associations for sporadic AD include Jakobsdottir et al. who detected an association in the *TM2D3* gene. Work in Drosophila suggest that the damaging effect of this variant is through the β-amyloid cascade [59]. Kunkle et al. used WES in a mixed ethnic population to search for rare variants leading to sporadic early onset AD. The authors observed associations with missense variants in *PSD2*, *TCIRG1*, *RIN3* and *RUFY1.* Interestingly, these genes function in clearance of cellular debris and unwanted proteins, including Aβ, through the endolysosomal transport pathway [51]. Le Guennec et al. studied sporaric early onset AD and found a rare recurrent microduplication, affecting the 17q21.31 locus (including the *CRHR1*, *MAPT*, *STH* and *KANSL1* genes) in four cases but not in healthy controls. An increase in *MAPT* (encoding tau protein) expression was observed in the affected individuals, and neuroimaging and cerebrospinal fluid biomarker profiles suggest the primary role of *MAPT* in disease development [60].

The majority of studies testing rare variation in sporadic AD have focused on identifying association with disease development. Ridge and colleagues adopted an innovative, pedigree-based approach to identify genetic variation that segregate with AD resilience. They studied “AD-resilient” individuals who had the high-risk *APOE* ε4 allele and were above 75 years of age without any signs of cognitive decline assessed clinically and compared them with relatives who developed AD. The rs142787485 variant in *RAB10* was shown to significantly associate with “AD-resilience”, which replicated in an independent sample. Furthermore, in cell models, the knockdown of *RAB10* led to a statistically significant decrease in Aβ42 and Aβ42/Aβ40 ratio [53].

It must be noted that these associations have largely been observed in Caucasian populations, However, the majority of non-Caucasian studies are underpowered [61]. The disparity of results in non-Caucasian studies of *TREM2* [26, 27, 36•, 38, 42–47], the work of Kunkle et al. [51] (detailed above), the cross-population and population-specific associations seen at *ABCA7* [62, 63] and the identification of putative association at the *AKAP9* gene in an African American cohort [64] emphasise the ethnic specific genetic aetiology of AD and the need for further research in this area.

## Methods for Rare Variant Discovery

The gold standard for rare variant discovery remains WGS, assaying every base in the genome. WGS allows the analysis of the full range of genomic modifications including pathogenic variants, structural variants and variants in non-coding regulatory regions [65–68]. Additionally, WGS is the superior method for covering difficult genomic regions including those with high GC content due to its PCR-free sequencing protocol. However, given the nature of rare variation, potentially being seen once in less than 1000 individuals, the sample sizes required to achieve statistical power for association analysis make this method of data generation economically prohibitive. The majority of the known risk loci were identified by genome-wide genotyping microarrays, including low frequency variants *PLCG2* and *ABI3*. These arrays only assay known genetic variation and despite imputation accuracy down to MAF = 0.008 when using the latest reference panels, a large proportion of low and rare frequency variants do not genotype or impute well on such arrays.

An alternative, and intermediate experiment between WGS and GWAS is WES. This method assays bases in the protein coding regions of the genome (the exome), meaning that any identified associations are likely to have an understandable functional effect. The exome makes up about 1% of the human genome, making WES a cheaper and popular alternative to WGS for both exonic and splice-site [69] rare variant detection. It has been estimated that the exome harbours about 85% of mutations with large effects on disease-related traits [70]. Exome sequencing studies have brought to light the importance of rare coding variants in complex genetic traits that were undetectable by GWAS [71]. Being a comprehensive approach, exome sequencing also provides direct identification of the casual variants, both common and rare, without the use of linkage disequilibrium to impute genotypes, as routinely performed with GWAS data. Exome sequencing was especially successful in the identification of Mendelian disease genes. This is reflected in almost 2000 new entries in OMIM since 2008 describing the genetic basis of a certain phenotype [70]. Therefore, the three primary advantages of exome sequencing over other rare variant detection methods are; the high potential to identify genes responsible for complex traits, readily available functional annotation of coding variants and the cost-effectiveness of WES compared to the WGS.

Several platforms for human exome capture are on the market [69, 72, 73]. The question of which of these platforms is best for a given application remains unanswered, as with any experimental technique, there are both strengths and limitations. The major difference between these platforms corresponds to the number of genes targeted, the probe/bait lengths, probe/bait density and sequencing coverage. There is also some difference in capture efficacy performance (including specificity, uniformity and sensitivity), technological reproducibility, DNA input requirement and cost effectiveness of each platforms. A comprehensive comparison of all the commercially available human WES platforms is beyond the scope of this review and has been performed elsewhere [69, 72, 73]. It has to be said that none of the capture technologies are able to cover all of the exons of the Consensus CDS, RefSeq or Ensembl databases.

## Challenges of Rare Variant Identification

Rare variant identification has proved challenging in common disease; there are a number of factors to explain this, including the method of variant detection and the methods of data merging. Microarrays assay only known variation meaning that a substantial proportion of rare risk loci are potentially missed. Additionally, calling and clustering of rare variants via standard GWAS techniques have proved to be problematic and labour intensive, with much of the Illumina exome chip content requiring visual inspection [36•]. WGS captures all bases in the genome, resulting in the generation of a large amount of data. This can prove both computationally challenging and difficult to interpret given a number of identified variants may lie outside regions of known functional relevance. Often WGS data are filtered to include only functionally relevant variation, such as protein-coding and splice site-specific variation, to reduce such burdens.

The majority of rare variant detection studies utilise, at least in part, WES. Assembly of WES data within an experiment is now relatively standardised, with specifically designed software, quality control and analysis pipelines [74]. However, as evidenced from meta-analysis of GWAS [7, 75], much of the power of genetic analysis of complex traits is gained through combination of data from multiple independent experiments. This can prove problematic for WES experiments where different capture technologies have been utilised. None of the capture technologies available are able to cover all of the exons of the Consensus CDS, RefSeq or Ensembl databases. Of the four commercially available human capture kits on the market (NimbleGen, Agilent, TruSeq and Nextera), only 26.2 Mb of the total targeted bases overlap, equating to around a 1/3 of the total targeted bases per kit (NimbleGen targets 64.1 Mb, Agilent targets 51.1 Mb, TruSeq and Nextera target 62.08 Mb). Therefore, combination of data generated via differing capture kits can result in a significant loss of target bases and subsequent lack of analysis of potential disease-related mutations. An additional technical issue with combination of WES data is the differing base coverage achieved by each capture kit. For the 26 Mb target regions, common to all four technologies, Agilent detected the highest number of variants followed by TruSeq, Nextera and NimbleGen [72]. Additionally, the areas of optimal coverage differ by capture technology, with the largest number of Illumina variants detected in the untranslated regions compared to NimbleGen detecting the highest number of variants in the Ensembl regions [72]. These findings emphasise the importance of sequence capture uniformity and capture probe performance, which eventually determine the amount of raw sequence data available for downstream data analysis. Ideally, all studies would use the same capture technology to allow merging of raw data for optimal rare variant detection.

Another intricacy, specifically in the analysis of rare variants, is that by definition, rare variants are not frequent and therefore association tests of individual variants is challenging [76]. The typical GWAS of common variants strategy is analysis of one variant at a time. Such analysis will be largely underpowered for rare variant detection unless the variant effect size or the sample size of the cohort is very large. This is why a number of methods have been developed to analyse multiple rare variants collapsed together thus increasing the statistical power [77–80]. An exhaustive review of how to design and analyse data based on rare variants is beyond the scope of this manuscript and is specific to the study design and technology utilised. A number of conceptual frameworks for the design of rare variant association studies have been published [76, 81, 82]. Rare variant analyses, whether at the single variant or gene-wide level, require large sample sizes to provide the required statistical power for the genetic association analyses. Zuk et al. have shown that the analysis of common variant and rare variant studies requires similarly large sample collections. In particular, a well-powered rare variant association study should involve discovery sets with at least 25,000 cases, together with a substantial replication set [76].

## Benefit of Rare Variant Discovery

**Fig. 1:**
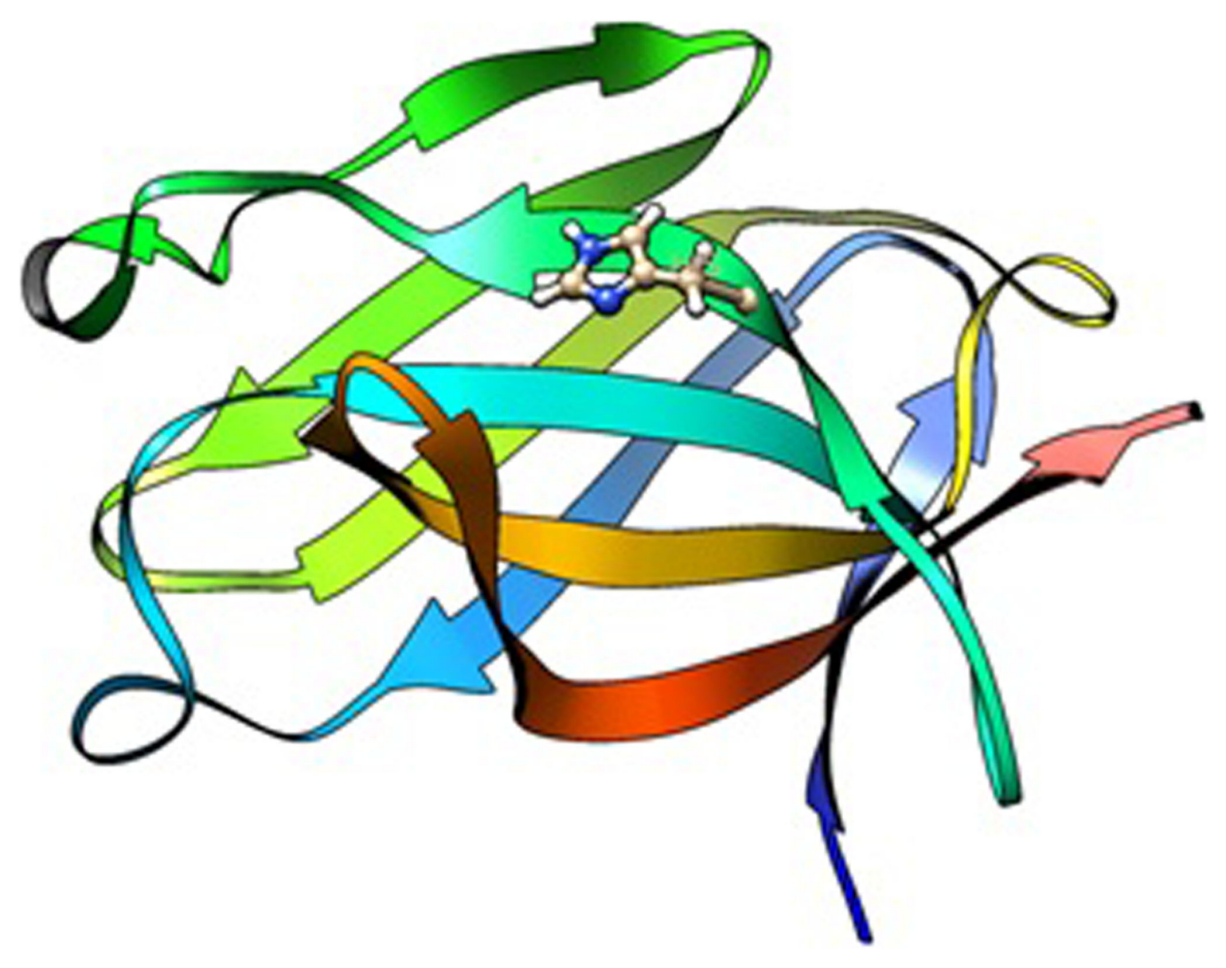
The binding domain of the TREM2 protein, the R62H rare variant is seen in a stick all ball model format

The single nucleotide rare variant associations identified are protein coding, meaning that the effect of the amino acid change can be easily modelled in silico and via cellular and animal models. This allows for a much quicker translation from genetics to a functional outcome and can be utilised in efforts to validate therapeutic targets [83]. Molecular dynamic (MD) modelling is an in silico technique that is gaining momentum in its use to understand rare variants in many diseases [84–88]. One example of the use of both cellular and in silico models to further our understanding of the impact rare variants have on AD is that of the well-studied TREM2 coding changes which have been subject of a number of in vitro, in vivo and in silico models [89–91]. Homozygous mutations within TREM2 leading to complete loss of function are a known cause of Nasu Hakola syndrome [92], which includes symptoms of frontotemporal dementia [93]. The identified AD-risk variants are thought to result in partial loss of function. The publication of purified proteins including that of TREM2 [91, 94], allows for in silico mutational studies into the impact of the variants on the protein structure and by inference, its function. Indeed, we and others have successfully modelled the TREM2 rare variants in silico with interesting results [91, 95]. Using MD simulations, we were able to conclude that the binding differences observed in vitro between the two genome wide significant mutations, R47H and R62H, could be attributed to a different structural alteration in their binding loops (Fig. 1) [95]. TREM2 has been shown to bind to apolipoproteins, including both APOE and CLU/APOJ and subsequently is involved in the uptake of Aβ by microglia. This may indicate that the different changes to the binding loop can be attributed to the variants differing genetic risk, and the differing binding rates as shown in vitro [96].

## Alternate Methods

It is becoming ever clearer that complex traits require more sophisticated data analysis methods to unpick the multifactorial aetiology of disease onset and progression. We know that AD is a polygenic disorder [6], simultaneous assessment of common and rare (i.e. polygenic and monogenic) models can be used to provide additional information about disease genetic architecture. This approach has been fruitful in studying blood lipid levels and neurodevelopmental disorders in large number of individuals [82, 97].

Instead of focusing on crude association studies, there are other innovative approaches that could provide additional information while studying rare variants. Some of the ways of exploring the data, include using other biological information, including gene expression (as reviewed by Verheijen and Sleegers [98]), methylation and biological pathways [99–101], in combination with genetic association data, to boost the statistical power of the analyses. To boost the statistical power of genetic association analyses, Ho et al. proposed a novel weightadjustment approach to combine gene expression, methylation, transcriptional regulation and protein abundance information into rare variant analysis. Simulation studies have suggested that incorporating together such rich data can lead to substantial gain in statistical power. This integrative approach was applied successfully to find susceptibility variants in genes associated with blood pressure regulation [78, 102]. Furthermore, a number of studies have successfully used similar methods to integrate GWAS data with biological networks data (protein-protein interaction and coexpression networks) to predict causal genes at associated GWAS loci for various disorders [103–106]. Such integrative approaches, albeit currently focused more on analysis of common variants, have proved successful in studies of AD [107, 108].

To interrogate data from transcriptome-wide association studies (TWAS) studies, a TWAS hub was recently developed (http://twas-hub.org). The hub allows searchable access to TWAS results from hundreds of complex traits and dozens of expression studies based on the methodology described initially in Gusev et al. [109].

To better understand the pathobiology of disease, another way forward is to study a small number of carefully selected families with multiple affected individuals and with strong family history. This analysis is likely to be successful given the risk variants are likely to have larger effect sizes than GWAS loci. In addition, because such variants are likely to be coding, it is easier to subsequently functionally characterise, and to develop cellular and animal models. Such approaches have been reviewed previously in Lord et al. [110]. In a similar vein, another study approach that has proved successful in finding novel risk genes for AD is to focus on early onset sporadic AD rather than late onset sporadic AD exemplified by Kunkle et al. and Nicolas et al. [51]. These studies focus on extreme phenotypes, likely to be enriched for rarer variants with moderate effect sizes. As sample sizes grow, identification of disease-modifying genetics-utilising cohorts with deeply phenotyped data is likely to prove fruitful to understand more about the genetics of disease progression. Individuals with AD experience a range of non-cognitive symptoms that are distinct to each individual with disease [111].

Another approach recently utilised to identify common risk variation is to analyse large data sets such as the UK Biobank. Although most of the participants in these types of studies are too young to be diagnosed with AD, it is possible to study the disorder using family history data via a diagnosis by family history design [21•, 112•]. Currently, the UK Biobank data only include a limited number of accurate rare variant genotype data. However, there are plans for the UK Biobank cohort to be sequenced and the data to be made available to the scientific community [113]. Further initiatives such as the Genomics England project and studies based on data from electronic health records could provide further opportunities to mine large sample sets of data [114]. A recent review discusses the available resources and the statistical challenges with respect to analysing such data [115]. In the UK, the newly announced Digital Innovation Hub Programme by Health Data Research UK (with the help of MRC) aims to build towards a national hub to connect health-related data for research across populations of between three to five million people [116]. A word of caution with respect to using primary health and longitudinal cohort data is the potential overlap of sample sets across multiple studies, which could lead to false-positive observations, and the continued requirement for independent replication of new loci in similar sized cohorts.

## Conclusions

Genetic heritability of sporadic AD is accounted for by both common and rare genetic variation. Here, we describe the established and putative rare AD risk variation identified by the field to date. We note that the rare variants identified contribute to disease susceptibility with larger effect sizes than generally seen with common risk variation and result in protein coding changes that can be easily modelled in silico, in vitro and in vivo. The identification of both common and rare disease-associated variants loci, including the *SORL1* and *ABCA7* genes, suggests that a number of the AD-associated genes may influence disease susceptibility via multiple mechanisms.

GWAS in other complex traits suggests that more powerful GWAS will identify further additional common and low frequency associations [25]. While, collaborative WES and WGS will undoubtedly unearth a significant number of rare variants that influence disease risk. As discussed, there are a number of issues that will need to be addressed to achieve this goal, primarily combining the differing sequence capture technologies, and adequately accessing the whole exome/genome. Further efforts by the IGAP, the European AD Biobank (EADB), AD European Sequencing (ADES) and ADSP among others are already underway. Ultimately, the future lies in WGS projects that will allow the detection and testing of the full range of genomic variation (including large structural alterations) with disease status, and this study design is being utilised for a range of rare diseases in projects such as Genomics England. Unfortunately, for complex traits that rely on large sample sizes to achieve the necessary statistical power, this is still beyond our reach.

The rare variants shown to associate with sporadic AD include potentially damaging and loss of function mutations, suggesting that careful assessment has to be considered for clinical practice and patient feedback along with the already established variation in *APOE*, *PSEN1*, *PSEN2* and *APP* [117]. Unquestionably, we are only at the beginning of our journey to identify rare protein-coding changes associated with disease. Already, the disease-associated protein-coded changes detected provide a greater understanding of the specific mechanism underlying disease risk as compared with common non-coding genetic risk factors and are likely to allow the expedited development of therapeutics.
